# Prevalence of Tuberculosis in Children After Natural Disasters, Bohol, Philippines

**DOI:** 10.3201/eid2510.190619

**Published:** 2019-10

**Authors:** Kristy O. Murray, Nina T. Castillo-Carandang, Anna M. Mandalakas, Andrea T. Cruz, Lauren M. Leining, Salvacion R. Gatchalian, on behalf of the PEER Health Bohol Pediatric Study Team

**Affiliations:** Baylor College of Medicine and Texas Children’s Hospital, Houston, Texas, USA (K.O. Murray, A.M. Mandalakas, A.T. Cruz, L.M. Leining);; University of the Philippines, Manila, Philippines (N.T. Castillo-Carandang, S.R. Gatchalian)

**Keywords:** pediatric tuberculosis, Philippines, cluster survey, prevalence, epidemiology, children, bacteria, tuberculosis and other mycobacteria, natural disasters

## Abstract

In 2013, a severe earthquake and typhoon affected Bohol, Philippines. To assess the postdisaster risk for emergence of *Mycobacterium tuberculosis* infection in children, we conducted a cross-sectional multistage cluster study to estimate the prevalence of tuberculin skin test (TST) positivity and tuberculosis (TB) in children from 200 villages in heavily affected and less affected disaster areas. Of the 5,476 children we enrolled, 355 were TST-positive (weighted prevalence 6.4%); 16 children had active TB. Fourteen (7%) villages had >20% TST-positive prevalence. Although prevalence did not differ significantly between heavily affected and less affected areas, living in a shelter with >25 persons approached significance. TST positivity was independently associated with older age, prior TB treatment, known contact with a person with TB, and living on a geographically isolated island. We found a high TST-positive prevalence, suggesting that national programs should consider the differential vulnerability of children and the role of geographically isolated communities in TB emergence.

In October 2013, the island province of Bohol, Philippines, was devastated by a 7.2-magnitude earthquake, followed 3 weeks later by the landfall of Typhoon Haiyan (Super Typhoon Yolanda). These disasters resulted in the deaths of 195 persons in the province; displacement of 30% of the 1.2 million-person population (*1*); and disruption of routine health services, including prevention and treatment services provided by the National Tuberculosis Program (*2*). After other natural disasters, infrastructure loss resulted in individual patients being contagious for longer periods, and increased *Mycobacterium tuberculosis* transmission occurred because of crowding in emergency shelters (*3*). In complex emergencies, children are the most vulnerable population and suffer the greatest negative effects (*4*). Approximately 400,000 children live in Bohol, so the increased risk for tuberculosis (TB) emergence after these natural disasters was expected to be substantial. To further complicate matters, the main island province of Bohol includes 75 smaller islands and islets that are considered geographically isolated and disadvantaged areas (*5*,*6*). These areas are separated from mainstream society and have both physical (i.e., accessible only by boat) and socioeconomic factors that further compound their vulnerability to TB.

In this study, our primary objectives were to estimate the prevalence of *M. tuberculosis* infection and TB disease between displaced and nondisplaced children and examine risk factors for *M. tuberculosis* infection. We aimed to clarify the epidemiology of childhood TB in the late postdisaster recovery setting and provide recommendations to mitigate damage and ensure preparedness before future complex emergencies.

## Methods

### Study Population

We conducted this study in the island province of Bohol in the Philippines during 2016–2018. Bohol is 4,821 km^2^ and comprises 1 city, 47 municipalities, and 1,109 villages (called barangays). In 2010, the total population of Bohol was ≈1,255,128, of whom 32% were children (*7*). The World Health Organization estimates that >80% of children are vaccinated with *M. bovis* BCG at birth in the Philippines (*8*).

### Study Design

To estimate the prevalence of tuberculin skin test (TST) positivity and TB in children (<15 years of age), we conducted a cross-sectional survey using a modified version of a multistage cluster sampling technique based on the World Health Organization’s Expanded Programme on Immunization coverage survey methods (*9*). Based on our initial sample size calculations, we determined that we needed to screen a minimum of 4,014 children (0–14 years of age) to identify a significant difference between our hypothesized postdisaster prevalence of *M. tuberculosis* infection (1%) and a reference value of 0.56% prevalence of infection (α = 0.05, power = 80%) (*10*). To account for the possibility of missing data or incomplete or inaccurate records, we aimed to sample 4,200 children.

Using 7 households per cluster and an estimated minimum average of 3 children per household, we determined we needed 200 clusters to obtain our sample size. The 200 clusters comprised 100 clusters chosen from the municipalities that suffered the greatest effects of the natural disasters (heavily affected areas) and 100 clusters from municipalities that suffered fewer effects (less affected areas) based on data from the Provincial Health Office (Reymoses Cabagnot, Provincial Health Officer, pers. comm., 2015 Aug 17). We randomly selected 7 municipalities each from heavily affected and less affected areas, providing 14 municipalities total for sampling.

To select the 200 clusters, we alphabetically arranged the names of all villages and their population sizes (based on the 2010 census), stratified by heavily affected area and less affected area designation. We determined the sampling interval by dividing the total population of each area (224,212 in heavily affected areas and 214,072 in less affected areas) by the number of clusters needed. We identified the first cluster (village) by using a randomly generated 5-digit number and matching it to the first village in our list with a cumulative population greater than or equal to the random number. We identified the second cluster by adding the sampling interval to the random number and selected subsequent clusters by adding the sampling interval to the previously generated number until we identified 100 clusters in each area (Appendix Tables 1, 2).

Once we identified all 100 clusters in each area, we selected the households for enrollment using simple random sampling in the field. We worked with the barangay health stations to obtain a list of all the households within the village, which we then randomly selected using a random number generator. The household number randomly drawn was the starting point of the survey. Each subsequent household was chosen by going to the next closest front door. If no one was home, then the next house was selected, until a total of 7 households containing >1 child were obtained for each of the 200 clusters (total households 1,400). All children within the household were enrolled.

**Figure 1:**
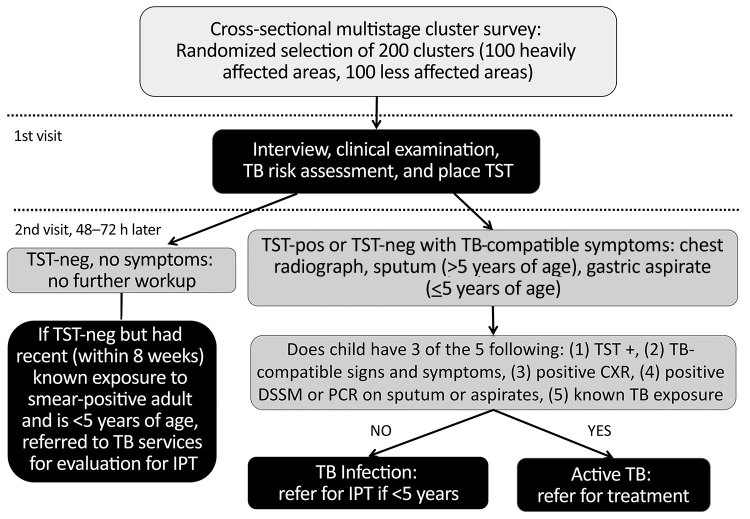
Procedures and decision tree for enrollment of study participants during community-based cluster survey of TB in children in areas affected by 2013 natural disasters, Bohol, Philippines. Positive result on chest radiograph means evidence of infiltrates, consolidation, or cavitary lesions suggestive of TB disease. DSSM, direct sputum smear microscopy; IPT, isoniazid preventive therapy; neg, negative; pos, positive; TB, tuberculosis; TST, tuberculin skin test.

All 1,400 households had an equal chance of being selected to participate in this survey. Children were excluded if caregivers did not provide consent or if child assent for those >7 years of age was not obtained. We conducted surveys using 2 questionnaires, 1 for the household in general and 1 for each child assessed. Surveys assessed social risk factors for *M. tuberculosis* infection, including whether or not the child was residing in Bohol during the disasters, displacement into an emergency shelter or camp, and number of new permanent or temporary residents in households who were displaced as a result of the disasters. We also assessed history of TB treatment and determined whether the children received their healthcare from the public or private sector. Caregivers completed screening for pulmonary TB using the National Tuberculosis Program questionnaire that assesses cough, weight loss, fever, and TB exposure (*11*); an examination for cervical lymphadenopathy (>2 × 2 cm); and TST (5 tuberculin units purified protein derivative–S, Serum Statens Institute, https://en.ssi.dk) (Figure 1).

### Clinical Evaluation for TB

The study team returned to each enrolled household 48–72 hours after the initial visit to measure the TST induration transversely in accordance with National Tuberculosis Control Program guidelines (*11*). All children who had TSTs >10 mm (or >5 mm if recent TB exposure within the last 6 months was known), had TB-compatible signs or symptoms, or both completed further evaluation for TB. Evaluation included physical examination, chest radiography, and microbiologic testing of sputum (children >5 years of age) or gastric aspirates (children <5 years of age) by direct smear sputum microscopy and GeneXpert PCR testing (Cepheid, http://www.cepheid.com); mycobacterial culture was not available. All TST-positive or symptomatic children were provided with transportation to the closest medical center along with a voucher for chest radiograph. An independent radiologist read the chest radiographs to determine the presence of lesions consistent with intrathoracic TB.

Participants in whom *M. tuberculosis* infection or TB disease were diagnosed was referred to the local health center for appropriate treatment. *M. tuberculosis* infection was defined as TST results >10 mm in asymptomatic children with normal chest radiograph results and negative direct smear sputum microscopy and PCR. In accordance with international and national guidelines (*11*,*12*), TB was diagnosed in children who met 3 of the 5 following criteria: 1) TST positive, 2) known exposure to a TB contact, 3) evidence of TB on chest radiograph, 4) direct smear sputum microscopy or PCR positive in sputum or gastric aspirates, and 5) 3 of the 6 signs and symptoms compatible with TB. Signs and symptoms of TB were cough or wheezing of >2 weeks, unexplained fever >2 weeks after common causes excluded, weight loss or failure to gain weight or weight faltering or anorexia, failure to respond to >2 weeks of antimicrobial therapy when treated for a lower respiratory tract infection, failure to return to baseline health status after >2 weeks after a viral infection or exanthema, and fatigue/lethargy or reduced playfulness (*11*). Participants in whom illnesses other than TB were diagnosed also were referred to the local health center for medical management. The Institutional Review Boards of the University of the Philippines Manila (Manila, Philippines) and Baylor College of Medicine (Houston, TX, USA) reviewed and approved this study.

### Data Analysis

All data were entered into EpiInfo version 7.2 (US Centers for Disease Control and Prevention, https://www.cdc.gov/epiinfo/index.html) on password-protected computers and were continuously backed up to a US-based protected server accessible only by study personnel. Statistical analyses were performed using EpiInfo and NCSS (NCSS, Inc., https://www.ncss.com). We determined the weighted prevalence of TST positivity (including diagnosed TB) and calculated Wilson 95% CIs. We then used univariate logistic regression with calculation of odds ratios (ORs) and 95% CIs to examine whether the prevalence of TST positivity in heavily affected areas differed significantly from that in less affected areas. We also performed univariate analysis on all other collected variables that could potentially influence the risk for TST positivity. Multivariate logistic regression analysis was then performed on all variables identified on univariate analysis with a p value <0.25 to determine independent risk factors for TST positivity in Bohol. We used a stepwise-backward approach to eliminate variables with the highest p value until all remaining variables had a p value <0.05. Model building strategies included interaction terms to determine effect modification and confounding.

## Results

During 2016–2018, a total of 5,476 children (2,710 in heavily affected areas and 2,766 in less affected areas) were enrolled from the 14 municipalities from the 184 villages selected for the 200 clusters. We enrolled an average of 3.9 children per household, exceeding our original sample size estimate of 3 children per household.

**Figure 2:**
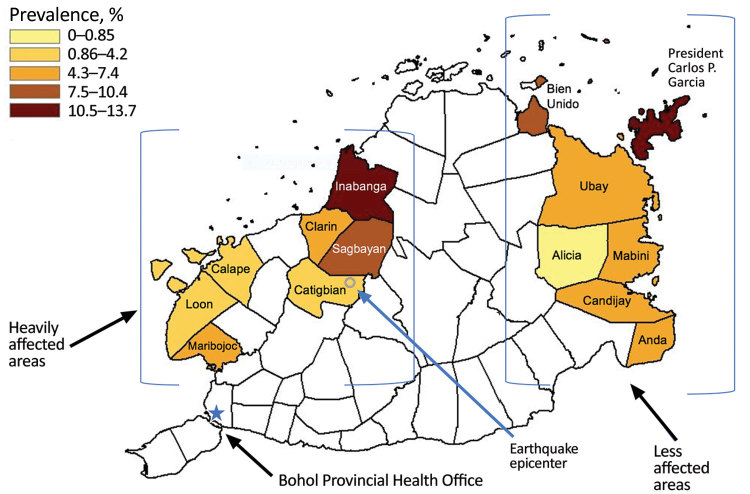
Prevalence of tuberculin skin test positivity by municipality obtained in study of tuberculosis in children in areas affected by 2013 natural disasters, Bohol, Philippines, 2016–2018. Epicenter of 2013 earthquake is indicated.

A total of 355 children were TST positive (weighted prevalence 6.4% [95% CI 6.3%–6.5%]). Three of the 14 municipalities had a TST-positive prevalence >10% (1 in heavily affected areas, 2 in less affected areas; Table 1, Figure 2), and 12 villages had TST-positive prevalence >20% (Appendix Table 3). Two remote villages (1 in heavily affected areas, 1 in less affected areas) had the highest prevalence (29% each). Of the 16 island villages located offshore from mainland Bohol, 9 (56%) had prevalence >10%, compared with 38 (22%) of the 168 villages on mainland Bohol.

**Table 1:** Prevalence of TST positivity by municipality and area affected by 2013 natural disasters, Bohol, Philippines, 2016–2018*

Municipality†	Total population† of municipality‡	Total no. children enrolled	Total no. TST positive§	Prevalence, % (95% CI)
Heavily affected area	199,653	2,710	160	5.9 (5.0–6.8)
Loon	42,729	550	14	2.5 (1.2–3.9)
Calape	30,146	260	11	4.2 (1.8–6.7)
Maribojoc	20,477	168	11	6.5 (2.8–10.3)
Clarin	20,277	267	16	6.0 (3.1–8.9)
Catigbian	22,675	624	19	3.0 (1.7–4.4)
Inabanga	43,272	537	62	11.5 (8.8–14.3)
Sagbayan	20,077	304	27	8.9 (5.7–12.1)

Less affected area	213,899	2,766	195	7.0 (6.1–8.0)
Ubay	68,482	653	48	7.4 (5.3–9.4)
Bien Unido	25,782	162	17	10.5 (5.7–15.3)
Pres. Carlos P. Garcia	23,269	212	29	13.7 (9.0–18.3)
Anda	16,866	327	21	6.4 (3.8–9.1)
Mabini	28,172	722	51	7.1 (5.2–8.9)
Candijay	29,043	457	27	5.9 (3.7–8.1)
Alicia	22,285	233	2	0.9 (−0.3–2.1)

*TST, tuberculin skin test. †Heavily affected and less affected areas each comprised 100 clusters/700 households. ‡Population is based on the 2010 national census (*7*). §TST positives include all tuberculosis cases, including the 1 child with tuberculosis who was TST negative because of malnutrition.

**Figure 3:**
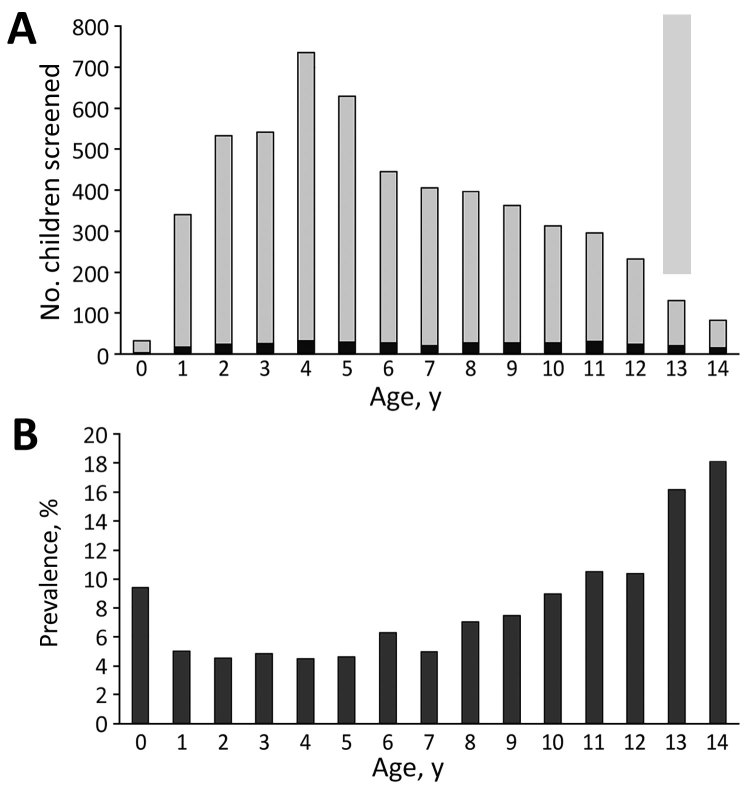
Distribution of patients by age in study of tuberculosis in children in areas affected by 2013 natural disasters, Bohol, Philippines. A) Number of children who screened positive by TST; B) prevalence of TST positivity. Black bars, TST positive; gray bars, TST negative. TST, tuberculin skin test.

Sex was not associated with TST positivity (Table 2). Older age was significantly associated with TST positivity; prevalence increased markedly (>10%) in children >10 years of age (Figure 3). Variables identified on univariate analysis as being significant risks for TST positivity were being older (>6 years of age), living in 1 of the island villages away from mainland Bohol, having a history of TB treatment, having >6 persons living in the home, having a history of contact with a person with TB, and having >2 weeks of cough during the preceding month.

**Table 2:** Demographic, social, and clinical histories of enrolled children in cluster survey of TB in children in areas affected by 2013 natural disasters, Bohol, Philippines*

Characteristic	Total, n = 5,476 (%)	TST		OR (95% CI)	p value
		Positive† n = 355	Negative, n = 5,121		
Male sex	2,862 (52.3)	179 (50.4)	2,684 (52.4)	1.1 (0.9–1.4)	0.44
Median age, y (IQR)	5.8 (5.3)	7.8 (6.3)	5.8 (5.2)		
0–5	2,811 (51.3)	133 (37.5)	2,678 (52.3)	Reference	
6–14	2,665 (48.7)	222 (62.5)	2,443 (47.7)	1.8 (1.5–2.3)	<0.001
Island village	375 (6.8)	48 (13.5)	327 (6.4)	2.3 (1.7–3.2)	<0.001
Prior treatment for TB	57 (1.0)	12 (3.4)	45 (0.9)	4.0 (2.1–7.6)	<0.001
Median no. persons living in household before earthquake (range)	5 (1–21)	6 (1–15)	5 (1–21)	1.1 (1.0–1.1)	0.009
>6 Persons living in home	2,586 (47.2)	193 (54.4)	2,393 (46.7)	1.4 (1.1–1.7)	0.005
Smokers in the home	3,049 (55.7)	208 (58.6)	2,841 (55.5)	1.1 (0.9–1.4)	0.23
Child had contact with person with TB	658 (12.0)	136 (38.3)	522 (10.2)	5.4 (4.3–6.8)	<0.001
Recent history of cough for >2 wk‡	104 (1.9)	26 (7.3)	78 (1.5)	4.9 (3.1–7.7)	<0.001

*All values are no. (%) unless indicated otherwise. IQR, interquartile range; OR, odds ratio; TB, tuberculosis; TST, tuberculin skin test. †TST-positive includes persons with TB. ‡Within 4 wk. Active represented 9 (35%) of the 26 TST-positive persons with a recent history of a cough for >2 wk.

Most (75.4%) of the children enrolled were already born and living in Bohol during the earthquake, and almost half (47.4%) were displaced. Among those in Bohol during the earthquake, living in a shelter with >25 persons approached significance for increased risk for TST positivity on univariate analysis (OR 1.5, 95% CI 0.98–2.2; p = 0.06). We noted no significant difference in TST positivity between heavily affected and less affected areas (Table 3). A higher proportion of TST-positive children were from the less affected areas, but this finding was not statistically significant.

**Table 3:** Factors related to 2013 earthquake and subsequent displacement on TST positivity in children in areas affected by 2013 natural disasters, Bohol, Philippines*

Factor	Total no. (%), n = 5,476	TST		Odds ratio (95% CI)	p value
		Positive, no. (%),† n = 355	Negative, no. (%), n = 5,121		
Earthquake-affected area					
Heavily affected area	2,710 (49.5)	160 (45.1)	2,550 (49.8)	1.2 (0.97–1.5)	0.09
Less affected area	2,766 (50.5)	195 (54.9)	2,571 (50.2)		
Child lived in Bohol during earthquake	4,131 (75.4)	278 (78.3)	3,853 (75.2)	1.2 (0.92–1.5)	0.20
Child was displaced	1,959/4,131 (47.4)	113/278 (40.6)	1,846/3,854 (47.9)	0.7 (0.6–0.95)	0.02
Child lived with ≥25 persons in shelter	1,081/1,959 (55.2)	72/113 (63.7)	1,009/1,846 (54.7)	1.5 (0.98–2.2)	0.06
Child displaced >7 d	777/1,956 (39.7)	50/113 (44.2)	727/1,843 (39.4)	1.2 (0.8–1.8)	0.31

*TST, tuberculin skin test.  †Includes persons with tuberculosis.

On the basis of results from the univariate analyses, we entered the following variables into the multivariate logistic regression model to determine which factors were independent risks for TST positivity: age category (6–14 years), history of TB treatment, prior contact with a person known to have TB, recent history of cough for >2 weeks, living on a remote island village, and living with >25 persons during displacement after the earthquake. Based on backward, stepwise multivariate logistic regression modeling, being older (OR 1.6; 95% CI 1.2–2.0), having a history of TB treatment (OR 3.4; 95% CI 1.7–6.7), contact with a person known to have TB (OR 4.9; 95% CI 3.8–6.2), and living on a remote island village (OR 1.5; 95% CI 1.1–2.1) were independent risk factors for TST positivity (Table 4).

**Table 4:** Independent risk factors for being TST positive in multivariate logistic regression analyses in cluster survey of TB in children in areas affected by 2013 natural disasters, Bohol, Philippines*

Variable	OR (95% CI)	p value
History of contact with a person known to have TB	4.9 (3.8–6.2)	<0.001
History of treatment for TB	3.4 (1.7–6.9)	<0.001
Older age, 6–14 y	1.6 (1.2–2.0)	<0.001
Living on a remote island village	1.5 (1.1–2.1)	0.02

*OR, odds ratio; TB, tuberculosis; TST, tuberculin skin test.

According to history provided by caregivers, 57 (1%) children were previously treated for TB; only 12 (22%) were TST positive (Table 2). We were unable to assess whether the treatment administered was isoniazid preventive therapy for TB exposure or latent infection or was treatment for active disease. Of the 57 reporting prior TB treatment, 47 (82%) completed the course of treatment, 8 (14%) did not complete treatment, and 2 (4%) had unknown treatment adherence. All 8 children who did not complete treatment were from villages that were hard to reach because of distance or accessibility. Reasons for not completing treatment were inability to purchase medications (5 children); erratic medicine supply (2 children); and distance from clinic, adverse medicine events, unpleasant taste, and difficult medication administration (1 child each). For 2 children, >1 barrier was listed for not completing treatment.

Intrathoracic TB was diagnosed in 16 (0.3%) children (median age 6 years) (Table 5). Three (24%) had microbiological confirmation (all by GeneXpert). Seven (44%) had abnormal radiographic findings consistent with TB. The most commonly reported history of recent (within 4 weeks) symptoms were cough >2 weeks (9 [56%] children) and weight loss/anorexia (6 [38%]). On physical examination, 7 (44%) children had cervical lymphadenopathy. All 16 children with TB were included in the total number of TST-positive children in the analyses to examine risks for exposure.

**Table 5:** Clinical and diagnostic findings for 16 persons with TB in cluster survey of TB in children in areas affected by 2013 natural disasters, Bohol, Philippines, 2016–2018*

Case no.	Natural disaster area	Age, y/sex	Known exposure to TB	History of signs/symptoms	Chest radiograph interpretation by radiologist	DSSM result†	GeneXpert result†
1	LAA	6/M	Yes	Cough >2 weeks, wheezing, weight loss; no improvement after taking antimicrobial drugs	Pneumonia, both paracardiac areas	Neg	Neg
2	LAA	2/M	Yes	Cough >2 weeks, weight loss, malaise; no improvement after taking antimicrobial drugs	Inflammatory process, both inner zones	Neg	Invalid, after 2 extractions
3	LAA	8/M	Yes	Cervical lymphadenopathy	Calcified hilar lymphadenopathy, likely representing a chronic process, such as pulmonary TB	Neg	Neg
4	LAA	14/M	Yes	None; history of prior TB treatment but did not complete therapy	Inflammatory process in left apical area compatible with chronic process, such as pulmonary TB with minimal apical pleural thickening	Neg	Neg
5	LAA	7/F	Yes	Cough >2 weeks	Normal	Neg	Neg
6	LAA	4/M	Yes	Cough >2 weeks, weight loss, anorexia, malaise, chest pain	Normal	Neg	Neg
7	LAA	5/M	Yes	Cervical lymphadenopathy	Inflammatory process in the left retrocardiac area	Neg	Neg
8	HAA	14/F	Yes	None	Normal	Neg	Pos
9	LAA	5/F	Yes	Cough >2 weeks, fever, weight loss	Normal	Neg	Pos
10	LAA	1/F	Yes	Cough >2 weeks, fever, dyspnea, no improvement after taking antimicrobial drugs	Normal	Neg	Neg
11	LAA	12/F	No	Coughing >2 weeks, fever, chest and back pain, weight loss, cervical lymphadenopathy	Normal	Neg	Neg
12	LAA	11/F	Yes	Cervical lymphadenopathy, no rales or wheezing	Normal	Neg	Neg
13	LAA	3/M	Yes	Cervical lymphadenopathy	Normal	Neg	Neg
14	LAA	6/M	Yes	Cervical lymphadenopathy	Normal	Neg	Neg
15	LAA	3/F	Yes	Coughing >2 weeks, weight loss	Bilateral pneumonia	Neg	Neg
16	HAA	10/M	Yes	Coughing >2 weeks, weight loss, cervical lymphadenopathy	Pneumonia, both lower lungs, minimal left pleural effusion vs. pleural thickening; consider Potts disease (extrapulmonary TB) involving T12 and L1 vertebrae with Gibbus deformity	Neg	Pos

*DSSM, direct sputum smear microscopy; HAA, heavily affected area; LAA, less affected area; neg, negative; pos, positive; TB, tuberculosis. †Direct smears and GeneXpert (Cepheid, http://www.cepheid.com) performed on sputum for children >5 years of age and gastric aspirates for children <5 years of age.

Among the 1,400 households in which we conducted interviews, 148 (11%) reported a household death within the 12 months before enrollment, including 10 deaths involving a family member with known or presumed TB. Among homes of TST-positive children, 17 deaths occurred in the previous year; 6 households reported death of a family member with known or presumed TB.

## Discussion

We assessed the risk for TB in a postdisaster setting among a large population of children using a methodologically rigorous study design. The prevalence of TST positivity was higher than we expected and disparate, even in a relatively small island province in the Philippines, and TST positivity in some villages approached 30%. Considering that the the weighted prevalence of TST positivity of 6.4% and that 422,148 children live in Bohol (*7*), we can estimate that ≈27,000 children are TST positive in this 1 province. At the time of this study, TST prevalence for children in the Philippines was unknown. Although we did not find TST positivity to be significantly higher in disaster-affected areas in Bohol as a result of resource interruptions as we originally hypothesized, positivity was associated with geographic barriers (i.e., island villages) and approached significance with increased risk resulting from crowding in emergency shelters. In adults, smear positivity and illness and death increased after natural and humanmade disasters in countries in Central America (*13*), Eastern Europe (*14*), and Africa (*15*). Our data add a perspective for children and are consistent with data reported for TB for adults in developing countries after complex humanitarian emergencies.

The high prevalence of TST positivity among subgroups of children in Bohol was unexpected. Unfortunately, we know of no prior studies in children in this region that would have enabled us to document baseline or estimate the expected prevalence. Villages with high prevalence of TST positivity might plausibly have unique risk factors for TB (e.g., geographically isolated and disadvantaged areas having poor socioeconomic status or limited access to care). TSTs also might have overestimated the incidence of *M. tuberculosis* infection resulting from cross-reactions with BCG (*16*). In the Philippines, BCG is administered only once, soon after birth (*17*), which provides a lower risk for false positive TSTs than in countries where BCG is boosted or administered to older children (*18*). Also, if cross-reactions were common, we would not have observed such variation in prevalence of TST positivity across Bohol, particularly in older children, which was the higher risk group.

Robust national and international data demonstrate that TB occurs in pockets of persons and varies substantially across geographic regions (*19*,*20*). Although some clustering of cases may be explained by underlying medical, social, or economic conditions (e.g., diabetes, socioeconomic status, and care access issues), explanations for clustering are not always evident. We found higher TST positivity in island villages where geographic barriers prevented immediate access to the municipal health units on mainland Bohol. Increasing distance from public healthcare facilities can result in diagnostic delays and missed diagnoses, particularly for TB, where control programs often use centralized models. Late disease detection in infectious adults has substantial implications for children, including increasing *M. tuberculosis* infection and missed opportunities for preventive health services, outreach, and public health intervention.

In our study, other factors independently associated with TST positivity included older age, history of contact with a person known to have TB, and history of TB treatment. These statistical findings were expected because older children have a longer possible period of exposure risk over the course of their childhood. Similarly, known contact with a person with TB and history of TB treatment would greatly influence TST positivity. Although our finding of higher TST-positive prevalence in less affected areas than in heavily affected areas was not significant, we did not expect to find it. We hypothesize this finding was because less affected areas were much farther from the Provincial Health Office, where TB resources are distributed to the entire province. This discrepancy is worth investigating further to understand whether availability and access to resources affects TB transmission in this region.

Historically, TB prevention and treatment efforts have focused on adults for epidemiologic, economic, and practical reasons. *M. tuberculosis*–infected children are reservoirs for future cases and transmitters of disease. Given their youth, children are less likely to experience adverse side effects of TB prevention treatment and experience greater long-term benefits than adults, presuming they are not reinfected by the original source. Additionally, in many developing nations, children account for nearly 50% of the population. Thus, changing the emphasis of treatment and prevention programs to be more inclusive of children is needed but requires modification in provider education, expansion of diagnostic tools, caregiver support, and more readily available access to child-friendly medication formulations.

During natural disasters, disruption of TB control poses a threat to both industrialized and resource-limited nations, as seen after the 2011 earthquake in Japan, the 2010 earthquake in Haiti, and the 2005 Hurricane Katrina in the United States (*15*,*21*,*22*). Experience has demonstrated that major impediments to successful reconstruction of TB services include mobile populations, destroyed infrastructure, and lack of coordination, leading to poor case detection and suboptimal TB control (*15*). Our findings suggest that displacement after natural disasters may increase the future risk for TB in affected communities. Because public health resources are often introduced into communities after disasters, we propose that the postdisaster recovery period might provide a unique window of opportunity to introduce interventions to sustainably improve TB control.

Our study had some limitations. Epidemiologic risk factors were family-reported and subject to recall bias, particularly because this study was conducted 3–5 years after the natural disasters. Crowding in shelters with nonrelatives might have resulted in underestimating TB contacts for children. Interferon γ-release assays were unavailable; some TST positivity might have resulted from cross-reaction from BCG. However, older children were significantly more likely than younger children to be TST positive, which would not be expected if TST positivity were due solely to BCG. Although we presume that BCG uptake is high according to national data, we did not collect vaccine status individually at enrollment. The unavailability of mycobacterial cultures potentially caused an underestimation of the TB prevalence. Unfortunately, the number of active TB cases was small, so we were concerned about performing and interpreting any statistical analyses for risk; however, when active cases were examined independently in our model, the risks remained the same for this group with the exception of older age. Our findings might not be generalizable to other disaster settings in less populated regions or in areas with lower baseline TB incidence.

In conclusion, in a large, community-based screening for *M. tuberculosis* infection in children <15 years of age in the Philippines, we found a high prevalence of TST positivity, especially in geographically isolated villages. We demonstrated the feasibility and highlight the importance of implementing active TB case-finding in a resource-poor setting despite population displacement and postdisaster service-line interruption. One step to bolster postdisaster mitigation is a strong baseline national TB program that includes local stakeholders (including not only healthcare workers but also community and government leaders), reaches marginalized populations, and considers the differential vulnerability of children before a disaster.

Appendix Additional methods and results for a study on the prevalence of tuberculosis in children after natural disasters, Bohol, Philippines.
