# Supplementary material for: Prevalence of Tuberculosis in Children After Natural Disasters, Bohol, Philippines
Source: Emerg Infect Dis. 2019 Oct;25(10):1884–92. doi: 10.3201/eid2510.190619 (PMC6759243; doi:10.3201/eid2510.190619)
Supplement: Appendix — Additional methods and results for a study on the prevalence of tuberculosis in children after natural disasters, Bohol, Philippines. [file 19-0619-Techapp-s1.pdf]

Article DOI: https://doi.org/10.3201/eid2509.190619

Prevalence of Tuberculosis in Children After Natural Disasters, Bohol, Philippines

Appendix

Appendix Table 1. Cluster assignments in the heavily affected area, Bohol, Philippines*

Barangay - MUNICIPALITY	Population/barangay	Cumulative populations	Cluster
Abucayan Norte - CALAPE	1,326	1,326	
Abucayan Sur - CALAPE	786	2,112	74
Agahay - MARIBOJOC	603	2,715	
Agsoso - LOON	254	2,969	
Alegria - CATIGBIAN	1,408	4,377	75
Aliguay - MARIBOJOC	859	5,236	
Ambuan - CATIGBIAN	1,259	6,495	76
Anislag - MARIBOJOC	1,004	7,499	
Anonang - INABANGA	721	8,220	77
Baang - CATIGBIAN	1,607	9,827	
Bacani - CLARIN	1,208	1,1035	78
Badbad Occidental - LOON	279	11,314	
Badbad Oriental - LOON	509	11,823	
Badiang - INABANGA	1,083	12,906	79
Bagacay Katipunan - LOON	189	13,095	
Bagacay Kawayan - LOON	427	13,522	
Bagacay Saong - LOON	137	13,659	
Bagtic - CATIGBIAN	1,123	14,782	80
Baguhan - INABANGA	541	15,323	
Bahan - INABANGA	464	15,787	
Bahi - LOON	367	16,154	81
Banahao - INABANGA	652	16,806	
Banlasan - CALAPE	755	17,561	
Bentig - CALAPE	1,797	19,358	82
Baogo - INABANGA	1,252	20,610	83
Basac - LOON	1,414	22,024	84
Basdacu - LOON	962	22,986	
Basdio - LOON	561	23,547	
Bayacabac - MARIBOJOC	1,601	25,148	85
Biasong - LOON	323	25,471	
Binogawan - CALAPE	466	25,937	
Bogtongbod - CLARIN	1,377	27,314	86
Bonbon - CALAPE	1,222	28,536	87
Bonbon - CLARIN	1,487	30,023	
Bongbong - CATIGBIAN	757	30,780	88
Bongco - LOON	328	31,108	
Bontud - CLARIN	456	31,564	
Bood - MARIBOJOC	475	32,039	
Buacao - CLARIN	797	32,836	89
Buangan - CLARIN	796	33,632	
Bugang - INABANGA	696	34,328	
Bugho - LOON	285	34,613	90
Busao - MARIBOJOC	587	35,200	
Cabacongan - LOON	1,080	36,280	91
Cabadug - LOON	231	36,511	
Cabawan - MARIBOJOC	1,516	38,027	
Cabayugan - CALAPE	880	38,907	92
Cabog - CLARIN	764	39,671	
Caboy - CLARIN	571	40,242	93
Cabudburan - CALAPE	548	40,790	
Cabug - LOON	185	40,975	

Barangay - MUNICIPALITY	Population/barangay	Cumulative populations	Cluster
Cagawasan - INABANGA	1,290	42,265	94
Cagayan - INABANGA	390	42,655	
Calangahan - SAGBAYAN	910	43,565	
Calayugan Norte - LOON	737	44,302	95
Calayugan Sur - LOON	538	44,840	
Calunasan - CALAPE	798	45,638	
Caluwasan - CLARIN	222	45,860	
Cambailan - CATIGBIAN	933	46,793	96
Cambaquiz - LOON	1,042	47,835	
Cambitoon - INABANGA	919	48,754	97
Camias - CALAPE	505	49,259	
Campatud - LOON	365	49,624	
Candaigan - LOON	477	50,101	
Candajec - CLARIN	932	51,033	98
Candavid - MARIBOJOC	541	51,574	
Candumayao - CATIGBIAN	1,680	53,254	99
Canguha - CALAPE	283	53,537	
Canhangdon Occidental - LOON	848	54,385	100
Canhangdon Oriental - LOON	549	54,934	
Canigaan - LOON	826	55,760	
Canlinte - INABANGA	276	56,036	
Canmaag - LOON	404	56,440	1
Canmano - SAGBAYAN	1,006	57,446	
Canmanoc - LOON	319	57,765	
Canmaya Centro - SAGBAYAN	1,317	59,082	2
Canmaya Diot - SAGBAYAN	1,161	60,243	
Cansuagwit - LOON	291	60,534	3
Cansubayon - LOON	505	61,039	
Cantam-Is Bago - LOON	284	61,323	
Cantaongon - LOON	970	62,293	
Cantam-Is Baslay - LOON	495	62,788	4
Cantoyoc - CLARIN	343	63,131	
Cantumocad - LOON	852	63,983	
Catagbacan Handig - LOON	994	64,977	5
Catagbacan Norte - LOON	1,186	66,163	
Catagbacan Sur - LOON	973	67,136	6
Catmonan - CALAPE	1,221	68,357	
Cuaming - INABANGA	2,826	71,183	7, 8
Causwagan Norte - CATIGBIAN	1,715	72,898	9
Cawayan - INABANGA	1,147	74,045	
Cogon Norte - LOON	1,907	75,952	10
Cogon Sur - LOON	425	76,377	
Cogon - INABANGA	865	77,242	11
Comaang - CLARIN	471	77,713	
Cuasi - LOON	1,115	78,828	12
Dagnawan - INABANGA	637	79,465	
Dagnawan - SAGBAYAN	525	79,990	
Dagohoy - INABANGA	1,310	81,300	13
Dait Sur - INABANGA	622	81,922	
Danahao - CLARIN	856	82,778	14
Datag - INABANGA	559	83,337	
Desamparados - CALAPE	880	84,217	
Dipatlong - MARIBOJOC	1,562	85,779	15
Fatima - INABANGA	721	86,500	
Genomoan - LOON	362	86,862	16
Guiwanon - MARIBOJOC	569	87,431	
Hagbuaya - CATIGBIAN	1,033	88,464	
Haguilanan - CATIGBIAN	1,184	89,648	17
Hambongan - INABANGA	523	90,171	
Ilaud - INABANGA	954	91,125	18
Ilaya - INABANGA	376	91,501	
Ilihan - INABANGA	398	91,899	
Jandig - MARIBOJOC	897	92,796	19
Kabasacan - SAGBAYAN	490	93,286	
Kagawasan - SAGBAYAN	370	93,656	
Kahayag - CALAPE	512	94,168	
Kang-Iras - CATIGBIAN	831	94,999	20
Katipunan - CLARIN	340	95,339	
Katipunan - SAGBAYAN	600	95,939	

Barangay - MUNICIPALITY	Population/barangay	Cumulative populations	Cluster
Kinabag-An - CALAPE	511	96,450	
Labuon - CALAPE	562	97,012	21
Lagtangon - MARIBOJOC	266	97,278	
Lajog - CLARIN	1,381	98,659	
Langtad - SAGBAYAN	570	99,229	22
Lapacan Norte - INABANGA	350	99,579	
Lapacan Sur - INABANGA	1,217	100,796	
Lawis - CALAPE	617	101,413	23
Lawis - INABANGA	1,389	102,802	
Libertad Norte - SAGBAYAN	316	103,118	24
Libertad Sur - CATIGBIAN	380	103,498	
Libertad Sur - SAGBAYAN	184	103,682	
Liboron - CALAPE	1434	105,116	25
Liboron - CATIGBIAN	1,349	106,465	
Liloan Norte - INABANGA	1,490	107,955	26
Liloan Sur - INABANGA	954	108,909	27
Lincod - MARIBOJOC	1,781	110,690	
Lintuan - LOON	913	111,603	28
Lo-oc - CALAPE	506	112,109	
Lomboy - CALAPE	490	112,599	
Lomboy - INABANGA	589	113,188	29
Lonoy Cainsican - INABANGA	656	113,844	
Lonoy Roma - INABANGA	593	114,437	
Looc - LOON	1,070	115,507	30
Lucob - CALAPE	1,330	116,837	
Lutao - INABANGA	1,173	118,010	31
Luyo - INABANGA	732	118,742	
Mabuhay - INABANGA	383	119,125	32
Madangog - CALAPE	622	119,747	
Magtongtong - CALAPE	404	120,151	
Mahayag Sur - CATIGBIAN	330	120,481	
Mahayag Norte - CATIGBIAN	722	121,203	33
Maitum - CATIGBIAN	1,035	122,238	
Mandaug - CALAPE	1,451	123,689	34
Mantalongon - SAGBAYAN	707	124,396	
Mantasida - CATIGBIAN	1,025	125,421	35
Mantatao - CALAPE	967	126,388	
Maria Rosario - INABANGA	424	126,812	
Mataub - CLARIN	700	127,512	36
Mocpoc Norte - LOON	875	128,387	
Mocpoc Sur - LOON	646	129,033	
Moto Norte - LOON	1,369	130,402	37
Moto Sur - LOON	1,225	131,627	38
Nabuad - INABANGA	1,804	133,431	39
Nagtuang - LOON	493	133,924	
Nahawan - CLARIN	2,208	136,132	40
Napo - INABANGA	706	136,838	
Napo - LOON	1,342	138,180	41
Nueva Vida - LOON	263	138,443	
Ondol - INABANGA	1,122	139,565	42
Pagnitoan - MARIBOJOC	630	140,195	
Panagquilon - LOON	496	140,691	
Pantudlan - LOON	808	141,499	43
Pig-Ot - LOON	592	142,091	
Poblacion Centro - CLARIN	1,234	143,325	44
Poblacion Norte - CLARIN	838	144,163	
Poblacion Sur - CLARIN	1,159	145,322	45
Poblacion Weste - CATIGBIAN	1,830	147,152	
Poblacion - CATIGBIAN	1,752	148,904	46
Poblacion - INABANGA	930	149,834	47
Poblacion - MARIBOJOC	2,298	152,132	48
Poblacion - SAGBAYAN	3,945	156,077	49, 50
Pondol - LOON	1,476	157,553	51
Punsod - MARIBOJOC	644	158,197	
Punta Cruz - MARIBOJOC	770	158,967	
Quinobcoban - LOON	185	159,152	
Riverside - INABANGA	260	159,412	52
Rizal - CATIGBIAN	770	160,182	
Saa - INABANGA	634	160,816	

Barangay - MUNICIPALITY	Population/barangay	Cumulative populations	Cluster
Sagbayan Sur - SAGBAYAN	1,011	161,827	53
Sampoangon - CALAPE	373	162,200	
San Agustin - SAGBAYAN	867	163,067	
San Antonio - SAGBAYAN	852	163,919	54
San Isidro - CALAPE	2,412	166,331	55
San Isidro - INABANGA	992	167,323	
San Isidro - MARIBOJOC	525	167,848	56
San Isidro - SAGBAYAN	736	168,584	
San Jose - INABANGA	1,566	170,150	57
San Ramon - SAGBAYAN	405	170,555	
San Roque - MARIBOJOC	1,177	171,732	58
San Roque - SAGBAYAN	420	172,152	
San Vicente Norte - SAGBAYAN	715	172,867	
San Vicente Sur - SAGBAYAN	290	173,157	
San Vicente - MARIBOJOC	1,115	174,272	59
Santa Catalina - SAGBAYAN	721	174,993	
Santa Cruz - CALAPE	2,401	177,394	60
Santa Cruz - SAGBAYAN	985	178,379	61
Santo Niño - INABANGA	799	179,178	
Santo Rosario - INABANGA	997	180,175	62
Sinakayanan - CATIGBIAN	881	181,056	
Sojoton - CALAPE	664	181,720	63
Sondol - LOON	690	182,410	
Song-On - LOON	683	183,093	
Sua - INABANGA	554	183,647	64
Talisay - CALAPE	415	184,062	
Talisay - LOON	1,310	185,372	
Tambook - INABANGA	490	185,862	65
Tan-Awan - LOON	110	185,972	
Tangaran - CLARIN	850	186,822	
Tangnan - LOON	867	187,689	66
Taytay - LOON	292	187,981	
Ticugan - LOON	373	188,354	
Tinibgan - CALAPE	733	189,087	
Tinibgan - MARIBOJOC	614	189,701	67
Tiwi - LOON	112	189,813	
Tontonan - LOON	607	190,420	
Tontunan - CLARIN	559	190,979	
Toril - MARIBOJOC	457	191,436	
Triple Union - CATIGBIAN	1,223	192,659	68
Tubod - CLARIN	402	193,061	
Tubodacu - LOON	395	193,456	
Tubodio - LOON	207	193,663	
Tubuan - LOON	285	193,948	69
Tultugan - CALAPE	830	194,778	
Tungod - INABANGA	1,089	195,867	70
U-Og - INABANGA	1,112	196,979	
Ubayon - LOON	635	197,614	
Ubojan - LOON	486	198,100	71
Ubojan - SAGBAYAN	988	199,088	
Ubujan - INABANGA	1,064	200,152	72
Ulbujan - CALAPE	1,445	201,597	
Villaflor - CLARIN	345	201,942	73

*Using a sampling interval of 2,019 (population of 201,942 divided by 100 clusters) and a random 5-digit generated number (56389) used as the starting point. Barangays (villages) were arranged in alphabetical order. Blank cells indicate locations not selected as cluster.

Appendix Table 2. Cluster assignments in the less affected area, Bohol, Philippines*

Barangay - MUNICIPALITY	Population/barangay	Cumulative populations	Cluster
Abaca - MABINI	2,782	2,782	63
Abad Santos - MABINI	814	3,596	
Abihilan – CANDIJAY	1,327	4,923	64
Achila – UBAY	1,276	6,199	65
Aguining - PRES. CARLOS P. GARCIA	2,294	8,493	66
Aguipo - MABINI	1,905	10,398	
Almaria - ANDA	392	10,790	67
Anoling - CANDIJAY	1,583	12,373	
Bacong - ANDA	2,289	14,662	68

Barangay - MUNICIPALITY	Population/barangay	Cumulative populations	Cluster
Badiang - ANDA	1,277	15,939	69
Basiao - PRES. CARLOS P. GARCIA	911	16,850	
Baud - PRES. CARLOS P. GARCIA	603	17,453	
Bay-Ang - UBAY	1,656	19,109	70
Baybayon - MABINI	1,886	20,995	71
Bayog - PRES. CARLOS P. GARCIA	309	21,304	
Benliw - UBAY	2,223	23,527	72
Biabas - UBAY	2,573	26,100	73
Bilangbilangan Dako - BIEN UNIDO	1,920	28,020	74
Bilangbilangan Diot - BIEN UNIDO	845	28,865	75
Bogo - PRES. CARLOS P. GARCIA	990	29,855	
Bonbonon - PRES. CARLOS P. GARCIA	1,286	31,141	76
Bongbong - UBAY	807	31,948	
Bood - UBAY	2,717	34,665	77
Boyo-An - CANDIJAY	1,632	36,297	78
Buenasuerte - ANDA	398	36,695	
Buenavista - UBAY	688	37,383	
Bulawan - MABINI	789	38,172	79
Bulilis - UBAY	1,711	39,883	
Butan - PRES. CARLOS P. GARCIA	626	40,509	80
Cabatang - ALICIA	675	41,184	
Cabidian - MABINI	1,348	42,532	81
Cadapdapan - CANDIJAY	1,605	44,137	
Cagongcagong - ALICIA	423	44,560	
Cagting - UBAY	1,597	46,157	82
Calanggaman - UBAY	1,623	47,780	83
California - UBAY	801	48,581	
Camali-An - UBAY	581	49,162	
Camambugan - UBAY	2,251	51,413	84
Cambane - CANDIJAY	665	52,078	85
Cambaol - ALICIA	1,087	53,165	
Campamanog - PRES. CARLOS P. GARCIA	1,560	54,725	86
Can-Olin - CANDIJAY	1,637	56,362	87
Canawa - CANDIJAY	2,466	58,828	88
Candabong - ANDA	2,297	61,125	89
Canmangao - PRES. CARLOS P. GARCIA	948	62,073	
Casate - UBAY	2,512	64,585	90
Casica - ANDA	406	64,991	
Cawayanan - MABINI	2,035	67,026	91
Cayacay - ALICIA	1,713	68,739	92
Cogtong - CANDIJAY	3,220	71,959	93, 94
Concepcion (Banlas) - MABINI	1,615	73,574	
Cuya - UBAY	516	74,090	95
Del Mar - MABINI	850	74,940	
Del Monte - ALICIA	806	75,746	
Fatima - UBAY	3,235	78,981	96, 97
Gabi - UBAY	1,378	80,359	
Gaus - PRES. CARLOS P. GARCIA	1,365	81,724	98
Governor Boyles - UBAY	888	82,612	
Guintabo-An - UBAY	686	83,298	99
Hambabauran - UBAY	1,106	84,404	
Hingotanan East - BIEN UNIDO	2,283	86,687	100
Hingotanan West - BIEN UNIDO	1,665	88,352	1
Humayhumay - UBAY	1,708	90,060	2
Ilihan -UBAY	802	90,862	
Imelda - UBAY	1,761	92,623	3
Juagdan - UBAY	1,121	93,744	
Kabangkalan - PRES. CARLOS P. GARCIA	309	94,053	
Katarungan - UBAY	1,524	95,577	4
Katipunan - ALICIA	2,230	97,807	5
Katipunan - ANDA	503	98,310	
La Hacienda - ALICIA	3,710	102,020	6, 7
La Union – CANDIJAY	1,691	103,711	8
Lapinig - PRES. CARLOS P. GARCIA	967	104,678	
Liberty - BIEN UNIDO	843	105,521	
Linawan – ANDA	987	106,508	9
Lipata - PRES. CARLOS P. GARCIA	685	107,193	
Lomangog - UBAY	2,025	109,218	10
Los Angeles - UBAY	436	109,654	

Barangay - MUNICIPALITY	Population/barangay	Cumulative populations	Cluster
Luan - CANDIJAY	937	110,591	11
Lundag - ANDA	1,029	111,620	
Lungsoda-An - MABINI	1,309	112,929	12
Lungsoda-An - CANDIJAY	1,853	114,782	13
Mahangin - CANDIJAY	1,059	115,841	
Mahayag - ALICIA	687	116,528	
Malingin - BIEN UNIDO	1,997	118,525	14
Mandawa - BIEN UNIDO	2,328	120,853	15, 16
Maomawan - BIEN UNIDO	1,475	122,328	
Marcelo - MABINI	1,167	123,495	
Minol - MABINI	1,721	125,216	17
Napo - ALICIA	1,255	126,471	18
Nueva Esperanza - BIEN UNIDO	2,205	128,676	19
Nueva Estrella - BIEN UNIDO	1,576	130,252	
Pag-Asa - UBAY	1,168	131,420	20
Pagahat - ALICIA	586	132,006	
Pagahat - CANDIJAY	556	132,562	
Panadtaran - CANDIJAY	1,511	134,073	21
Panas - CANDIJAY	1,705	135,778	22
Pangpang - UBAY	1,220	136,998	
Paraiso - MABINI	773	137,771	23
Pinamgo - BIEN UNIDO	2,177	139,948	24
Poblacion I - MABINI	1,679	141,627	
Poblacion II - MABINI	2,068	143,695	25
Poblacion - ALICIA	4,064	147,759	26, 27
Poblacion - ANDA	1,295	149,054	28
Poblacion - BIEN UNIDO	3,082	152,136	29
Poblacion - CANDIJAY	4,320	156,456	30, 31
Poblacion - PRES. CARLOS P. GARCIA	2,700	159,156	32
Poblacion - UBAY	3,633	162,789	33, 34
Popoo - Pres. Carlos P. Garcia	977	163,766	
Progreso - ALICIA	1,019	164,785	35
Puerto San Pedro - BIEN UNIDO	1,137	165,922	
Putlongcam - ALICIA	1,578	167,500	36
Sagasa - BIEN UNIDO	1,308	168,808	
Saguise -PRES. CARLOS P. GARCIA	745	169,553	37
San Francisco - UBAY	1,677	171,230	
San Isidro - CANDIJAY	1,380	172,610	38
San Isidro - MABINI	1,803	174,413	39
San Isidro - UBAY	707	175,120	
San Jose - MABINI	1,848	176,968	40
San Jose - PRES. CARLOS P. GARCIA	1,109	178,077	
San Pascual - UBAY	3,127	181,204	41, 42
San Rafael - MABINI	847	182,051	
San Roque - MABINI	2,981	185,032	43, 44
San Vicente - PRES. CARLOS P. GARCIA	893	185,925	
San Vicente - UBAY	1,074	186,999	
Santa Cruz - ANDA	1,123	188,122	45
Santo Rosario - PRES. CARLOS P. GARCIA	475	188,597	
Sentinila - UBAY	969	189,566	46
Sinandigan - UBAY	1,874	191,440	
Suba - ANDA	1,125	192,565	47
Sudlong - ALICIA	648	193,213	
Talisay - ANDA	1,048	194,261	48
Tambo - MABINI	958	195,219	
Tambongan - CANDIJAY	1,830	197,049	49
Tangkigan - MABINI	1,788	198,837	50
Tanod - ANDA	487	199,324	
Tapal - UBAY	1,371	200,695	
Tapon - UBAY	2,481	203,176	51, 52
Tawid - ANDA	825	204,001	
Tawid - CANDIJAY	1,129	205130	
Tilmobo - PRES. CARLOS P. GARCIA	197	205,327	53
Tintinan - UBAY	623	205,950	
Tipolo - UBAY	2,456	208,406	54
Tubod - CANDIJAY	2,052	210,458	55
Tubong - UBAY	885	211,343	
Tuboran - BIEN UNIDO	955	212,298	56
Tuboran - UBAY	1,372	213,670	

Barangay - MUNICIPALITY	Population/barangay	Cumulative populations	Cluster
Tugas - CANDIJAY	1,214	214,884	57
Tugas - PRES. CARLOS P. GARCIA	756	215,640	
Tugnao - PRES. CARLOS P. GARCIA	1,309	216,949	58
Union - UBAY	2,332	219,281	59
Untaga - ALICIA	1,804	221,085	
Valaga - MABINI	1,010	222,095	60
Villa Milagrosa - PRES. CARLOS P. GARCIA	1,273	223,368	61
Villa Teresita - UBAY	1,407	224,775	
Virgen - ANDA	1,428	226,203	62

*Using a sampling interval of 2,262 (population of 226,203 divided by 100 clusters) and a random 5-digit generated number (87628) used as the starting point. Barangays were arranged in alphabetical order. Blank cells indicate locations not selected as cluster.

Appendix Table 3. Prevalence of TST positives by each barangay (village) sampled, Bohol, Philippines*

Municipality	Barangay	TST positive	Total	Prevalence, %
Inabanga	Anonang	7	24	29
**Pres. Carlos P. Garcia**	**Gaus**	**6**	**21**	**29**
Inabanga	Dagohoy	6	22	27
Ubay	Cagting	6	23	26
Ubay	Sentinila	6	23	26
Inabanga	Badiang	6	25	24
**Pres. Carlos P. Garcia**	**Campamanog**	**5**	**22**	**23**
Sagbayan	Santa Cruz	12	53	23
**Inabanga***	**Cuaming**	**11**	**49**	**22**
**Bien Unido***	**Bilangbilangan Diot**	**5**	**23**	**22**
Inabanga	Sua	5	23	22
Bien Unido	Nueva Esperanza	5	24	21
Inabanga	Mabuhay	4	21	19
**Pres. Carlos P. Garcia**	**Poblacion**	**4**	**21**	**19**
**Pres. Carlos P. Garcia**	**Tugnao**	**4**	**21**	**19**
Maribojoc	Bayacabac	3	21	14
**Pres. Carlos P. Garcia**	**Butan**	**3**	**21**	**14**
Clarin	Candajec	4	28	14
Inabanga	Riverside	1	7	14
Inabanga	Tambook	3	21	14
Ubay	Union	3	21	14
Loon	Genomoan	3	22	14
Ubay	Imelda	3	22	14
Ubay	Katarungan	3	22	14
Anda	Bacong	5	38	13
Inabanga	Cagawasan	3	23	13
Ubay	Cuya	3	23	13
Ubay	Fatima	6	46	13
Clarin	Poblacion Centro	3	23	13
Catigbian	Poblacion	6	47	13
Inabanga	Cambitoon	3	24	13
Inabanga	Cogon	3	24	13
Candijay	Luan	3	24	13
Candijay	Tugas	3	24	13
Sagbayan	Canmaya Centro	6	49	12
Inabanga	Lomboy	2	18	11
Ubay	Tapon	5	46	11
Mabini	Cabidian	5	47	11
Candijay	Lungsoda-an	5	48	10
Mabini	Paraiso	4	40	10
**Pres. Carlos P. Garcia**	**Aguining**	**2**	**21**	**10**
**Pres. Carlos P. Garcia**	**Bonbonon**	**2**	**21**	**10**
Maribojoc	Dipatlong	2	21	10
Maribojoc	Jandig	2	21	10
Calape	Liboron	2	21	10
Maribojoc	San Vicente	2	21	10
Bien Unido	Tuboran	2	21	10
Mabini	Minol	5	54	9
Anda	Badiang	2	22	9
Inabanga	Santo Rosario	2	22	9
Calape	Sojoton	2	22	9
Mabini	Tangkigan	5	57	9
Ubay	Pag-asa	2	23	9
Bien Unido	Mandawa	4	47	9

Municipality	Barangay	TST positive	Total	Prevalence, %
Candijay	Poblacion	4	47	9
Calape	Abucayan Sur	2	24	8
Clarin	Caboy	2	24	8
Loon	Canhangdon Occidental	2	24	8
**Loon***	**Looc**	**2**	**24**	**8**
Calape	Mandaug	2	24	8
Candijay	Panadtaran	2	24	8
Anda	Talisay	2	24	8
Loon	Ubojan	2	24	8
Anda	Virgen	3	37	8
Ubay	Casate	2	25	8
Calape	Labuon	2	25	8
Clarin	Mataub	2	25	8
Clarin	Nahawan	2	25	8
Candijay	Panas	2	25	8
Candijay	Tubod	2	25	8
Mabini	Valaga	4	50	8
Inabanga	Liloan Sur	2	26	8
Mabini	Bulawan	3	41	7
Catigbian	Alegria	3	42	7
Mabini	San Isidro	3	43	7
Mabini	Poblacion II	7	101	7
Candijay	Cogtong	3	46	7
Ubay	Poblacion	3	46	7
Anda	Linawan	3	47	6
Anda	Candabong	2	32	6
Mabini	San Roque	4	64	6
Mabini	Baybayon	3	49	6
Mabini	Cawayanan	3	49	6
Mabini	San Jose	2	34	6
Sagbayan	Poblacion	3	52	6
Sagbayan	Sagbayan Sur	3	52	6
Sagbayan	Langtad	2	35	6
Anda	Santa Cruz	2	36	6
Ubay	Humayhumay	2	42	5
Inabanga	Liloan Norte	1	21	5
Ubay	Lomangog	1	21	5
Inabanga	Lutao	1	21	5
**Loon***	**Pantudlan**	**1**	**21**	**5**
**Pres. Carlos P. Garcia**	**Saguise**	**1**	**21**	**5**
Maribojoc	San Isidro	1	21	5
Maribojoc	San Roque	1	21	5
**Pres. Carlos P. Garcia**	**Villa Milagrosa**	**1**	**21**	**5**
Inabanga	Baogo	1	22	5
Candijay	Cambane	1	22	5
**Pres. Carlos P. Garcia**	**Tilmobo**	**1**	**22**	**5**
Clarin	Villaflor	1	22	5
Ubay	Biabas	1	23	4
Inabanga	Ilaud	1	23	4
Loon	Moto Sur	1	23	4
Mabini	Abaca	2	47	4
Ubay	Bay-ang	1	24	4
Calape	Cabayugan	1	24	4
Loon	Cogon Norte	1	24	4
Catigbian	Kang-iras	2	48	4
Loon	Lintuan	1	24	4
Loon	Tangnan	1	24	4
Clarin	Bacani	1	25	4
Bien Unido	Poblacion	1	25	4
Clarin	Poblacion Sur	1	25	4
Candijay	San Isidro	1	25	4
Catigbian	Ambuan	2	52	4
Alicia	Katipunan	1	27	4
Candijay	Tambongan	1	27	4
Anda	Poblacion	1	31	3
Catigbian	Causwagan Norte	1	33	3
Catigbian	Candumayao	1	35	3
Sagbayan	San Antonio	1	35	3
Anda	Suba	1	35	3

Municipality	Barangay	TST positive	Total	Prevalence, %
Ubay	San Pascual	1	44	2
Alicia	La Hacienda	1	45	2
Catigbian	Haguilanan	1	46	2
Mabini	Lungsoda-an	1	46	2
Catigbian	Triple Union	1	54	2
Catigbian	Mahayag Norte	1	55	2
Catigbian	Cambailan	1	58	2
Candijay	Abihilan	0	25	0
Ubay	Achila	0	26	0
Anda	Almaria	0	25	0
Catigbian	Bagtic	0	47	0
Loon	Bahi	0	21	0
Calape	Bentig	0	24	0
Loon	Basac	0	21	0
Ubay	Benliw	0	21	0
**Bien Unido***	**Bilangbilangan Dako**	**0**	**22**	**0**
Clarin	Bogtongbod	0	23	0
Calape	Bonbon	0	24	0
Catigbian	Bongbong	0	55	0
Ubay	Bood	0	23	0
Candijay	Boyo-an	0	23	0
Clarin	Buacao	0	22	0
Loon	Bugho	0	21	0
**Loon***	**Cabacongan**	**0**	**24**	**0**
Ubay	Calanggaman	0	22	0
Loon	Calayugan Norte	0	23	0
Ubay	Camambugan	0	21	0
Candijay	Canawa	0	23	0
Loon	Canmaag	0	25	0
Candijay	Can-olin	0	24	0
Loon	Cansuagwit	0	24	0
Loon	Cantam-is Baslay	0	24	0
Loon	Catagbacan Handig	0	21	0
Loon	Catagbacan Sur	0	24	0
Alicia	Cayacay	0	37	0
Loon	Cuasi	0	22	0
Clarin	Danahao	0	25	0
Ubay	Guintabo-an	0	45	0
Candijay	La Union	0	25	0
Calape	Lawis	0	23	0
Sagbayan	Libertad Norte	0	28	0
Catigbian	Mantasida	0	52	0
Loon	Moto Norte	0	24	0
Inabanga	Nabuad	0	24	0
Alicia	Napo	0	30	0
Loon	Napo	0	21	0
Inabanga	Ondol	0	21	0
Alicia	Poblacion	0	46	0
Inabanga	Poblacion	0	21	0
Maribojoc	Poblacion	0	21	0
Loon	Pondol	0	23	0
Alicia	Progreso	0	24	0
Alicia	Putlongcam	0	24	0
Calape	San Isidro	0	24	0
Inabanga	San Jose	0	8	0
Calape	Santa Cruz	0	25	0
Maribojoc	Tinibgan	0	21	0
Ubay	Tipolo	0	21	0
Loon	Tubuan	0	22	0
Inabanga	Tungod	0	24	0
Inabanga	Ubujan	0	23	0

*TST, tuberculin skin test. Bold indicates barangays on remote island areas separate from mainland Bohol.
